# Rare or Overlooked? Structural Disruption of Regulatory Domains in Human Neurocristopathies

**DOI:** 10.3389/fgene.2020.00688

**Published:** 2020-07-20

**Authors:** Víctor Sánchez-Gaya, Maria Mariner-Faulí, Alvaro Rada-Iglesias

**Affiliations:** ^1^Institute of Biomedicine and Biotechnology of Cantabria (IBBTEC), Consejo Superior de Investigaciones Científicas-University of Cantabria-Sociedad para el Desarrollo de Cantabria, Santander, Spain; ^2^Center for Molecular Medicine Cologne (CMMC), University of Cologne, Cologne, Germany; ^3^Cluster of Excellence Cellular Stress Responses in Aging-Associated Diseases (CECAD), University of Cologne, Cologne, Germany

**Keywords:** neural crest, structural variant, neurocristopathy, 3-D genome, enhancers, long-range regulation

## Abstract

In the last few years, the role of non-coding regulatory elements and their involvement in human disease have received great attention. Among the non-coding regulatory sequences, enhancers are particularly important for the proper establishment of cell type–specific gene-expression programs. Furthermore, the disruption of enhancers can lead to human disease through two main mechanisms: (i) Mutations or copy number variants can directly alter the enhancer sequences and thereby affect expression of their target genes; (ii) structural variants can provoke changes in 3-D chromatin organization that alter neither the enhancers nor their target genes, but rather the physical communication between them. In this review, these pathomechanisms are mostly discussed in the context of neurocristopathies, congenital disorders caused by defects that occur during neural crest development. We highlight why, due to its contribution to multiple tissues and organs, the neural crest represents an important, yet understudied, cell type involved in multiple congenital disorders. Moreover, we discuss currently available resources and experimental models for the study of human neurocristopathies. Last, we provide some practical guidelines that can be followed when investigating human neurocristopathies caused by structural variants. Importantly, these guidelines can be useful not only to uncover the etiology of human neurocristopathies, but also of other human congenital disorders in which enhancer disruption is involved.

## Enhancers Control the Establishment of Cell Type–Specific Gene-Expression Programs

For many years, most of the efforts to understand the human genome were focused on the study and annotation of coding sequences as this could potentially uncover the genetic basis of human disease. However, coding sequences only represent about 2% of the human genome (Elgar and Vavouri, 2008) although many of the remaining sequences are involved in gene regulation (ENCODE Project Consortium, 2012). Gene expression depends on regulatory sequences that act in *cis* (at the same chromosome) and respond to different factors (classically, transcription factors and long non-coding RNAs) that are codified by genes acting in *trans* (at different chromosomes) (Savarese and Grosschedl, 2006). The most relevant types of non-coding *cis*-regulatory sequences include promoters, enhancers, silencers, and insulators (Ong and Corces, 2011; Wittkopp and Kalay, 2012). Promoters are bound by a core set of widely used and highly conserved transcriptional regulators (e.g., RNA polymerase II, general transcription factors or GTFs, etc.) that confer basal transcriptional activity and enable transcription initiation (Brown and Feder, 2005). In contrast, enhancers positively control the expression of their target genes in time and space (Wray, 2007) and are major determinants of cell type–specific gene-expression programs (Bulger and Groudine, 2010, 2011; Buecker and Wysocka, 2012). Likewise, silencers and insulators also contribute to the establishment of specific gene-expression programs by repressing genes or blocking enhancers, respectively (Gaszner and Felsenfeld, 2006; Doni Jayavelu et al., 2020; Ngan et al., 2020; Pang and Snyder, 2020). The importance of non-coding regulatory sequences is well illustrated by the fact that up to 90% of the disease-associated variants reside in non-coding sequences, preferentially within putative enhancers (Maurano et al., 2012; Krijger and De Laat, 2016).

Despite the major regulatory functions of enhancers, their identification was historically a difficult task as they lack strong genetic-defining features (Elgar and Vavouri, 2008). However, it has been found that epigenomic profiling and chromatin signatures can be used as powerful and universal tools to identify enhancers (Heintzman et al., 2009; Rada-Iglesias and Wysocka, 2011; Rada-Iglesias et al., 2011). In particular, active enhancers are characterized by the binding of common coactivators (e.g., p300), an open chromatin conformation, the expression of short bidirectional RNAs (eRNAs), and by being flanked by nucleosomes marked with H3K4me1 and H3K27ac (Heintzman et al., 2009; Rada-Iglesias and Wysocka, 2011; Lam et al., 2014).

Enhancers can be located at great distances from their target genes. This is well exemplified by the *Sonic hedgehog* (*Shh*) locus, where an extensively studied enhancer, named ZRS, is located at the intron of a non-target gene (Lmbr1), approximately 850 kilobases away from *Shh*. The ZRS enhancer specifically controls the expression of *Shh* in the developing limb, and consequently, the disruption of this enhancer leads to severe limb malformations (Lettice, 2003; Sagai et al., 2005). In addition, enhancers sometimes skip their most proximal genes while controlling the expression of more distally located ones (Sanyal et al., 2012). As a consequence, it is difficult to assign enhancers to their target genes. However, the study of the three dimensional (3-D) structure of the DNA has contributed to overcome these limitations.

## Long-Range Gene Expression Control: Far in the Genome but Close in Nuclear Space

Enhancers can control the expression of genes located at very large genomic distances (i.e., long-range regulation) (Kleinjan and van Heyningen, 2005; Sagai et al., 2005). Here we focus on enhancer regulation in *cis*, which, at least in vertebrates, seems to be the most prevalent regulatory mechanism. Nevertheless, enhancer regulation can also occur in *trans*, with some interesting examples of interchromosomal enhancer–gene interactions being described in both flies and mammals (Müller and Schaffner, 1990; Bashkirova and Lomvardas, 2019; Monahan et al., 2019). Although several mechanisms have been proposed to explain the long-range regulatory activity of enhancers, the most accepted one is the so-called looping model, whereby enhancers and their targets become close to each other in 3-D nuclear space due to the formation of chromatin loops (Palstra et al., 2003).

**FIGURE 1:**
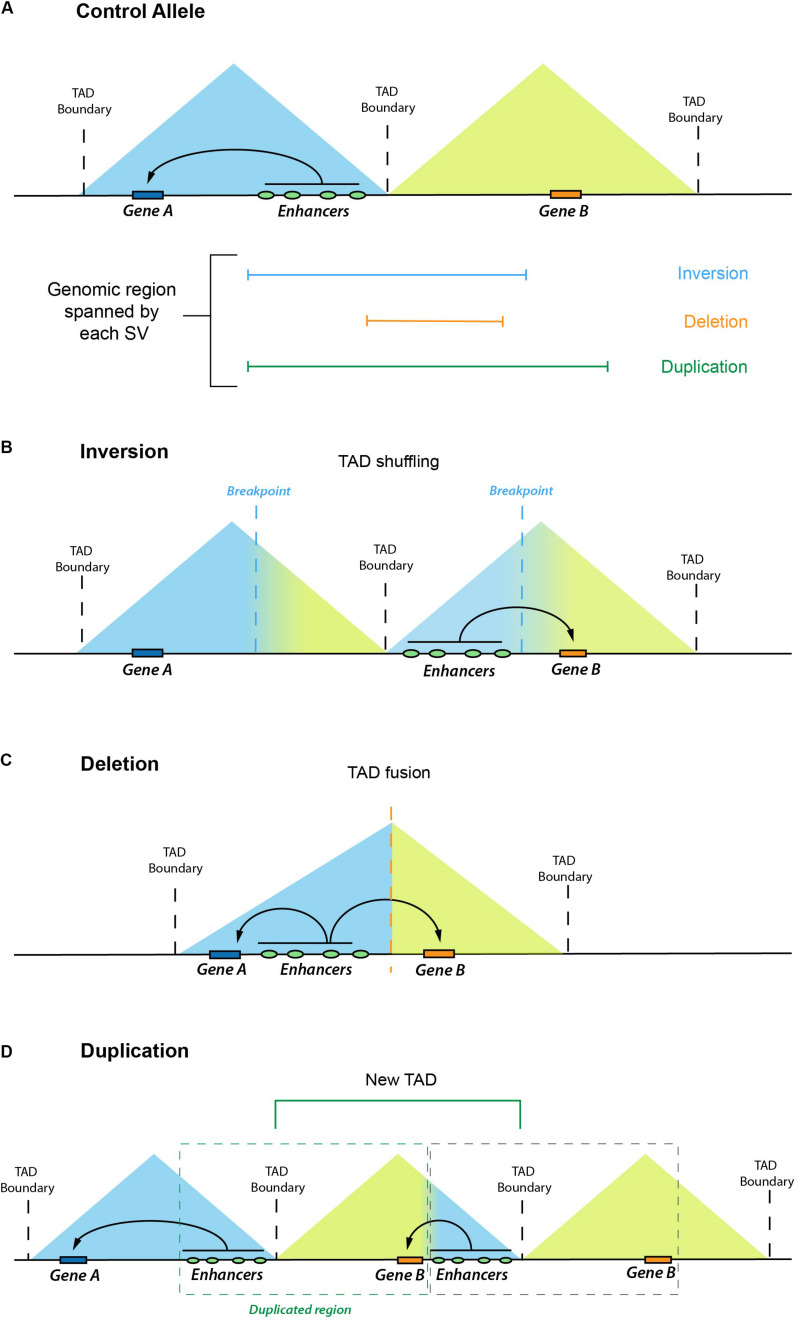
Enhancer adoption as a result of different structural variants (SV). Graphical overview of how different types of SV can lead to enhancer adoption mechanisms. In the control allele **(A)**, Gene A and Gene B are located in two neighboring TADs. In **(B–C)**, we illustrate how different genomic rearrangements, such as inversions **(B)**, deletions **(C)**, or duplications **(D)**, can remodel the 3-D chromatin landscape (through processes, such as TAD shuffling, TAD fusion, or formation of a new TAD, respectively) and increase Gene B expression due to the regulatory effects of ectopic enhancers.

The emergence of chromosome conformation capture (3C) techniques [e.g., Hi-C, 4C-seq, HiChip (Lieberman-Aiden et al., 2009; van de Werken et al., 2012; Mumbach et al., 2016)] has largely improved the study of 3-D genome organization and our capacity to systematically link enhancers with their target genes (Bickmore, 2013). These methods are based on the quantification of interaction frequencies between loci that lie in close spatial proximity independently of their linear genomic distance. One of the most relevant findings coming from studies using 3C-related methods is that genomes tend to be organized in megabase-scale regulatory domains named topologically associating domains (TADs) (Dixon et al., 2012). TADs contain genomic regions that interact with themselves with high frequency while interacting less often with the rest of the genome. The majority of the enhancer–gene interactions occur within TADs (Dixon et al., 2012; Nora et al., 2013; Rao et al., 2014; Spielmann et al., 2018). Moreover, TADs constrain the genomic regions that an enhancer can act upon and, thus, insulate enhancers from contacting ectopic target genes located in different TADs (Lupiáñez et al., 2016). The regions preventing contact between neighboring TADs are called boundaries or borders, which are preferentially bound and established by architectural proteins, such as CTCF and Cohesin (Dixon et al., 2012; Rao et al., 2014). Thus, TADs can be considered as fundamental regulatory units that facilitate enhancer–gene interactions within a domain while insulating regulatory activity from neighboring domains (Figure 1A). As we discuss in the following section, these concepts of 3-D genome organization have dramatically improved our capacity to predict and interpret the pathological consequences of human structural variation (SV).

## Pathological Disruption of Regulatory Domains by Structural Variants

**FIGURE 2:**
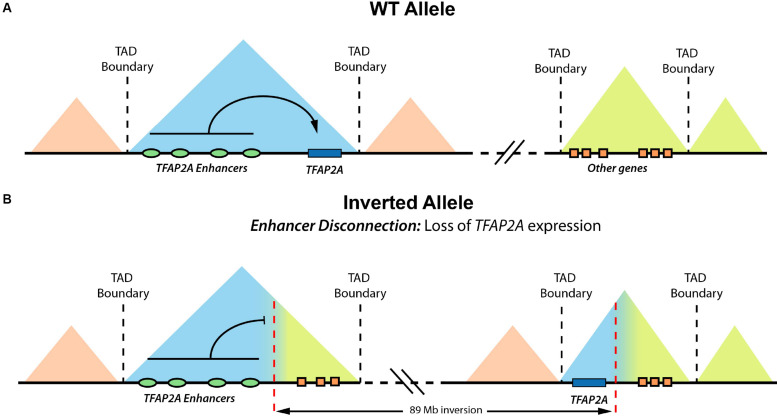
Enhancer disconnection in a reported BOFS patient. Graphical overview of how an heterozygous inversion found in a BOFS patient causes the physical disconnection between *TFAP2A* and its enhancers, leading to haploinsufficient *TFAP2A* expression in human neural crest cells (hNCCs). **(A)** Schematic representation of the wild-type (WT) *TFAP2A* allele. **(B)** Schematic representation of the altered *TFAP2A* allele, where an inversion disconnects *TFAP2A* from its cognate enhancers. Modified from Laugsch et al. (2019).

Structural variation (SV) refers to genomic alterations, including deletions, duplications, inversions, insertions, and translocations, that can largely differ in their sizes, ranging from a few base pairs (∼50 bp) to several megabases (Ho et al., 2020). Germline SVs are a common cause of congenital disease (Sebat et al., 2007; Walsh et al., 2008; Xu et al., 2008; Cooper et al., 2011; Soemedi et al., 2012), and high levels of somatic SVs are a key signature of human cancer genomes (Yang et al., 2013; Sudmant et al., 2015). There are different possible scenarios by which SVs can cause disease. The most studied and best understood SVs are those that directly affect coding sequences and, for example, delete, duplicate, or fuse genes. However, in many other cases, the pathogenic effect of SVs might involve changes in enhancer–gene communication, whose effects on gene expression can be best understood if 3-D genome architecture and TAD organization are taken into consideration. This topic has received great attention, and we direct the reader to some excellent reviews for more details (Krijger and De Laat, 2016; Lupiáñez et al., 2016; Spielmann et al., 2018). Briefly, to illustrate how SV can disrupt gene expression control, we discuss one example in which an inversion brings enhancers from the *EPHA4* TAD to the vicinity of *WNT6*, located in a neighboring TAD (Lupiáñez et al., 2015). This inversion causes ectopic interactions between *WNT6* and the *EPHA4* enhancers, leading to a pathological gain of *WNT6* expression in the developing limb and severe limb malformations. This type of pathological mechanism whereby enhancers cause the ectopic expression of non-target genes is known as “enhancer adoption” or “enhancer hijacking” (Lettice et al., 2011). Furthermore, the same inversion causes a loss of interactions between *EPHA4* and its cognate enhancers (i.e., “enhancer disconnection”), leading to *EPHA4* repression in the developing limb. Although, in this particular case, the loss of *EPHA4* expression is not responsible for the limb malformations, there are known instances in which SV can have pathological consequences due to similar “enhancer disconnection” mechanisms (Laugsch et al., 2019). In Figures 1, 2, we graphically illustrate how different SVs can lead to enhancer adoption or enhancer disconnection mechanisms. In addition to enhancers, silencer elements are also involved in the establishment of cell type–specific gene-expression programs. Although silencers have been historically difficult to identify and characterize at a mechanistic level, several recent reports indicate that silencers are abundant in mammalian genomes (Gaszner and Felsenfeld, 2006; Doni Jayavelu et al., 2020; Ngan et al., 2020; Pang and Snyder, 2020). Moreover, these studies also indicate that at least some silencers repress gene expression by physically interacting with their target genes (Gaszner and Felsenfeld, 2006; Doni Jayavelu et al., 2020; Ngan et al., 2020; Pang and Snyder, 2020). Therefore, similarly to enhancers, the disruption of silencers or silencer–gene communication can also contribute to disease. Overall, SVs can cause disease by disrupting TAD 3-D architecture and, consequently, the communications between enhancers/silencers and genes without altering the gene or enhancer/silencer sequences.

Different *in silico* methods and guidelines have been developed to predict and interpret the pathogenic effect of SV (Ibn-Salem et al., 2014; McLaren et al., 2016; Ganel et al., 2017; Weischenfeldt et al., 2017; Zepeda-Mendoza et al., 2017; Bianco et al., 2018; Yauy et al., 2018; Middelkamp et al., 2019; Hertzberg et al., 2020). Whereas some of these tools, such as SVScore (Ganel et al., 2017) or the Ensembl Variant Effect Predictor (McLaren et al., 2016), are restricted to gene direct effects or do not consider the patient-specific phenotype, others (Ibn-Salem et al., 2014; Zepeda-Mendoza et al., 2017; Middelkamp et al., 2019) are specifically designed to handle changes on gene–enhancer communication as well as to consider the patient’s particular phenotype. Briefly, these enhancer-gene–oriented approaches typically use TAD coordinates to delimit the genomic regions and genes that could be affected by SVs due to long-range regulatory effects. Subsequently, changes in the enhancer landscape caused by the SVs are analyzed, assessing whether any of the candidate genes could be subject to either a pathological gain (“enhancer adoption”) or loss (“enhancer disconnection”) of function. In addition, genes predicted to become silenced due to “enhancer disconnection” can be further prioritized by mining known gene–phenotype relationships from databases, such as OMIM^1^. Among them, genes previously associated with the patient phenotypes due to coding mutations or deletions would represent the strongest candidates. Furthermore, even if an SV directly affects a gene, this does not necessarily imply any pathogenic consequence, which may instead be caused by long-range regulatory changes in the expression of other gene/s. For instance, in the case of the *SHH* locus, deleting *LMBR1* would cause a limb malformation. However, this would not be due to the loss of *LMBR1* function, but rather due to the loss of the ZRS enhancer (located at an intron of *LMBR1*), which, as previously described, controls *SHH* expression in the limb. Taking these notions into account, some *in silico* approaches (Ibn-Salem et al., 2014; Middelkamp et al., 2019) estimate that a considerable fraction (10–30%) of the congenital abnormalities present in patients with SVs are caused by long-range regulatory mechanisms, either on their own or together with the direct disruption of protein-coding genes. These results, together with the fact that most disease-associated variants are found within putative enhancers (Maurano et al., 2012) emphasize the relevance and usefulness of these *in silico* tools. Nevertheless, and as we more extensively discuss in the following section, these predictions must be taken with caution because the regulatory rules dictating the compatibility between genes and enhancers seem to be more complex than previously anticipated.

## Enhancer Responsiveness: Being in the Same TAD Is Not Always Enough

Together with 3C technologies, the development of novel genetic engineering approaches, especially the CRISPR-Cas technique, is allowing us to dissect regulatory domains with unprecedented depth and resolution. Overall, the emerging picture is that the regulatory rules governing the compatibility between genes and enhances are rather complex (Arnold et al., 2017), and simply being in the same TAD is not sufficient for functional gene–enhancer interactions to take place (Ghavi-Helm, 2019). For example, the loss of CTCF or Cohesin function in mammalian cells results in an almost complete elimination of TAD boundaries, yet this has rather subtle effects on gene expression (Nora et al., 2017; Rao et al., 2017). Similarly, the structural disruption of TAD organization in Drosophila results in moderate gene-expression changes (Ghavi-Helm et al., 2019). Most recently, work from the PCAWG Consortium shows that only 14% of TAD boundary deletions found in human tumors resulted in significant changes in the expression of nearby genes (Akdemir et al., 2020). Nevertheless, the previous findings do not necessarily imply that TADs are not functional (Galupa et al., 2020), but rather that additional regulatory layers also contribute to the specific and functional communication between genes and enhancers. These additional layers are still largely unknown, but recent studies are starting to shed some light on this relevant topic. Using multiplex reporter assays, the Stark lab has demonstrated that distinct types of gene promoters largely differ in their enhancer responsiveness, which seems to depend, at least partly, on the cofactors that are bound to them (Arnold et al., 2017; Haberle et al., 2019). In another study, the Mundlos lab showed that, upon placing a cluster of enhancers in a novel TAD with multiple genes, those whose promoters were marked with H3K27me3/Polycomb responded more strongly to the enhancers (Kraft et al., 2019). In addition, extensive rearrangements within the *Shh* locus showed that altering the distance between *Shh* and its limb-specific enhancer ZRS does not apparently matter as long as both remain within the same TAD. However, when placing an insulator/barrier between *Shh* and the ZRS enhancer, reducing the distance enables *Shh*, at least partly, to recover its ZRS-dependent expression (Symmons et al., 2016). This last observation, together with another report in which the regulatory elements controlling *Xist* expression have been dissected (Galupa et al., 2020), suggest that TAD boundaries are not impenetrable, but rather partially permeable barriers that do not completely insulate genes from regulatory elements located in neighboring TADs. Moreover, TAD boundary positions are not strictly conserved among all cell types, and thus, future efforts should include obtaining Hi-C maps in additional cell types and tissues (Dixon et al., 2012; McArthur and Capra, 2020). Together with cell type–specific gene-expression data, these additional Hi-C maps would help improve the predictions of how SV might affect gene expression through long-range regulatory mechanisms. Last but not least, it is important to consider that many genes display complex regulatory landscapes in which multiple enhancers control in a totally or partially redundant manner the expression of their target genes (Osterwalder et al., 2018; Ghavi-Helm, 2019). Therefore, predicting the effects that the partial loss of the enhancers controlling a particular gene might have can be challenging.

In summary, to predict the long-range regulatory effects that a given SV might have, we should ideally consider not only TADs, but also the expression patterns of the candidate genes as well other regulatory factors, such as the type of gene promoters, the distance between genes and enhancers and the complexity of the affected enhancer landscapes. Since all these regulatory layers contributing to enhancer–gene communication are not fully understood, it is very important to experimentally validate the functional consequences of medically relevant SV.

## Long-Range Regulatory Effects in Neural Crest Cells as an Etiological Mechanism for Human Neurocristopathies

To further illustrate the pathological relevance of SV and of long-range gene regulation, we now focus on human neurocristopathies (NCP), a group of disorders characterized by congenital malformations in anatomical structures, such as the skeletal components of the head, the heart, or the peripheral nervous system (Simões-Costa and Bronner, 2013). NCP are caused by defects occurring during neural crest (NC) development, which, consequently, represents an obvious and appropriate model for the study of these human disorders. The NC is a vertebrate-specific embryonic cell population that originates in the dorsal neural tube. Once specified, the NC progenitors undergo an epithelial-to-mesenchymal transition and acquire impressive migratory capacity. Based on their anterior–posterior origin within the neural tube, the NC is divided into four different types: cranial, vagal, trunk, and sacral. The cranial NC specifically contributes to the development of the craniofacial skeleton, several anterior structures of the eye, teeth, and cranial ganglia. The vagal NC participates in the formation of the smooth muscle of the great vessels, the cardiac septa and the enteric ganglia. The trunk NC contributes to the development of the dorsal root ganglia, the sympathetic ganglia, and the adrenal medulla. Last, the sacral NC contributes to the proper development of the enteric ganglia (Simões-Costa and Bronner, 2013). Hence, the NC cells (NCCs) contribute to the morphogenesis and function of many different organs and tissues in the vertebrate body. Given the remarkable differentiation potential of the NC, it is not surprising that human NCP include many and diverse congenital abnormalities. However, and as we describe below, despite the prevalence of human NCP, the importance of non-coding pathological mechanisms in general and of long-range regulatory mechanisms in particular has not been extensively explored for this group of disorders.

Among the congenital abnormalities associated with human NCP, craniofacial malformations are particularly prevalent and can be found in more than 700 different syndromes (Trainor, 2010). Moreover, approximately one third of all newborns with congenital anomalies display head and face alterations, which represent a primary cause of infant mortality (Trainor, 2010). Among the NC-related craniofacial malformations, orofacial clefts are the most common ones, with a prevalence of 1 in 800 live births worldwide (Rahimov et al., 2012). The high incidence of craniofacial abnormalities is also seen among patients with congenital anomalies carrying SVs: 140 out of 273 patients (51.3%) described by Redin et al. (2017) display head, neck, or craniofacial defects. In addition to craniofacial malformations, which are mostly caused by defects during cranial NC development, human NCP include a broad range of abnormalities in other tissues and organs due to defects in other NC types (see Vega-Lopez et al. (2018) for an extensive review of NCP). For example, heterotaxy syndrome, a condition that causes a complex congenital heart disease, can be triggered by alterations in cardiac NCCs. On the other hand, congenital central hypoventilation syndrome (CCHS) is caused by impairments in the trunk NCC, and it is characterized by autonomic nervous system defects, shallow breathing, and the development of tumors (e.g., neuroblastoma). Last, one example of a syndrome caused by defects in cranial NCCs is branchio-oculo-facial syndrome (BOFS), which is characterized by several facial, ocular, hearing, and cutaneous anomalies. Previous studies show that BOFS is caused by heterozygous mutations or deletions that alter the coding sequence of the *TFAP2A* gene, which encodes for a transcription factor considered a NC master regulator (Milunsky et al., 2011, 2008). Interestingly, we recently described a BOFS patient who, in contrast to all previously reported cases, had two intact *TFAP2A* alleles. Instead, this patient presented a long heterozygous inversion that led to the physical disconnection between one of the *TFAP2A* alleles and its cognate NC enhancers, which resulted in *TFAP2A* monoallelic and haploinsufficient expression in cranial NCC (Laugsch et al., 2019; Figure 2). Hence, although this patient is still a rather isolated case, it illustrates that SV can cause NCP through long-range regulatory mechanisms.

In addition to the unique BOFS patient explained above, previous studies have described genetic changes in non-coding regulatory sequences as the possible cause for various NCP (Amiel et al., 2010). The mutation of an enhancer located at the first intron of the *RET* gene was associated with Hirschsprung disease susceptibility (Emison et al., 2005), an NCP caused by the failure of enteric NCCs to colonize the intestine (Vega-Lopez et al., 2018). Pierre Robin sequence (PRS), a neurocristopathy caused by abnormal cranial NCC development and characterized by craniofacial alterations, has been associated with deletions and point mutations of enhancers surrounding *SOX9* (Benko et al., 2009). Moreover, in addition to coding mutations within *SOX10* and other genes involved in Waardenburg syndrome, this human NCP might also be caused by alterations in enhancers surrounding *SOX10* (Bondurand et al., 2012; Lecerf et al., 2014). It is worth noting that the functional characterization and pathological relevance of these previously studied enhancers and the mutations therein were largely based on reporter assays. Although these assays provide important information about the enhancer activity of a given DNA sequence, they do not directly address whether an enhancer (and mutations therein) contribute to the expression of its predicted target gene and, thus, to the etiology of the associated human disorder. For instance, a single nucleotide polymorphism (SNP) that falls in an enhancer can alter its activity by affecting a transcription factor binding site, which can be detected by reporter assays. However, this SNP might not affect the expression of the predicted target gene due to compensatory effects of redundant enhancers (Osterwalder et al., 2018) or, considering the difficulties to assign enhancers to their target genes, might even control the expression of some other gene. The emergence of novel genome editing techniques, such as CRISPR-Cas, can largely overcome these limitations as endogenous enhancer loci can be genetically modified with high efficiency. In addition, many of the previous studies used animal models, such as mice, zebrafish, or chicken, which have been historically essential to molecularly characterize the neural crest and to dissect its gene regulatory networks (Sauka-Spengler and Bronner-Fraser, 2008; Green et al., 2015). However, when it comes to human congenital disorders in general and human NCP in particular, model organisms do not always faithfully recapitulate the phenotypes observed in human patients (Mestas and Hughes, 2004). On the other hand, it is important to mention that, in addition to enhancers, silencers should also be considered when investigating the role of non-coding regulatory sequences in human NCP (Doni Jayavelu et al., 2020; Ngan et al., 2020; Pang and Snyder, 2020). For instance, using insertional mutagenesis in mice, silencer elements contributing to the inactive state of Fam162b (Bergeron et al., 2015) and *Nr2f1* (Bergeron et al., 2016) in NCC were identified. Notably, disruption of those silencers relieved the repression of *Fam162b* and *Nr2f1* in NCC, which ultimately caused the emergence of phenotypes resembling those observed in Hirschsprung’s disease (Bergeron et al., 2015) and Waardenburg syndrome (Bergeron et al., 2016), respectively.

## Using *In Vitro*-Derived Human Neural Crest Cells to Model Neurocristopathies: Methods, Advantages, and Limitations

Model organisms, especially mice, have been extensively used to investigate the etiological mechanisms of human disease. Mouse models, in particular, offer a set of optimized and robust genetic and molecular tools that can be used to investigate developmental processes and/or disease progression in an *in vivo* context. Consequently, work in mice and other animal models (e.g., zebrafish, chicken) has been essential to understand complex developmental and morphological processes, such as those that occur during neural crest and craniofacial development and that get disrupted in human NCP (Sauka-Spengler and Bronner-Fraser, 2008; Cordero et al., 2011). However, there are important differences between mice and humans (Mestas and Hughes, 2004), for example, in gene dosage sensitivity: For many developmental genes implicated in human congenital disorders (including NCP), humans, but not mice, are haploinsufficient. This is well illustrated by BOFS: In humans this NCP is caused by heterozygous mutations/deletions in *TFAP2A*, and *Tfap2a*^+/–^ mice appear as morphologically normal (note that *Tfap2a*^–/–^ display a severe BOFS-like phenotype) (Schorle et al., 1996; Zhang et al., 1996; Brewer et al., 2004; Milunsky et al., 2011, 2008; Leblanc et al., 2013; Li et al., 2013). Therefore, when these differences in gene dosage sensitivity are encountered, the pathological mechanisms of congenital disorders should be ideally investigated in human cellular models. In addition, working with adult tissues to study congenital disorders is not fully appropriate because gene regulatory programs significantly differ between embryonic and adult stages, not to mention the even higher differences that exist between different or unrelated cell types/tissues. Taking all this into consideration, human NCC represent a relevant model to investigate human NCP.

However, having access to NCC is not easy due to their embryonic and migratory nature, which makes their isolation a difficult task. This is especially problematic in humans due to obvious ethical restrictions that limit the accessibility to human embryos. To overcome these limitations, several labs have established robust *in vitro* differentiation protocols that allow us to obtain NCC from human embryonic stem cells (hESC) or human induced pluripotent stem cells (hiPSC) (Bajpai et al., 2010; Menendez et al., 2013; Mica et al., 2013; Prescott et al., 2015; Fattahi et al., 2016; Huang et al., 2016; Hackland et al., 2017; Tchieu et al., 2017; Frith et al., 2018; Laugsch et al., 2019). These differentiation protocols can be broadly divided into those involving an intermediate embryoid body step and those in which the hESC/hiPSC are more directly differentiated into NCC. Each type of differentiation has its own advantages and disadvantages. For example, passing through an embryoid body step recapitulates important stages of NC differentiation, such as the epithelial-to-mesenchymal transition (EMT), whereby neural crest progenitors delaminate from the dorsal neural tube. On the other hand, more direct NCC differentiation protocols are faster and result in more homogenous cell populations. Regardless, these methods have proven to be useful to study both human NC development as well as the pathomechanisms of human NCP (Bajpai et al., 2010; Menendez et al., 2013; Mica et al., 2013; Prescott et al., 2015; Fattahi et al., 2016; Huang et al., 2016; Hackland et al., 2017; Tchieu et al., 2017; Frith et al., 2018; Laugsch et al., 2019). Nevertheless, these NC *in vitro* differentiation systems have some obvious and important limitations since the complexity and precision of *in vivo* embryogenesis can not be fully recapitulated, especially the morphogenesis of complex NCC-derived structures (e.g., palate) or the interactions that NCC established with their surrounding during embryo development. Therefore, *in vitro*-derived hNCCs do not represent the only or most appropriate model to study human NCP. Instead, each model has certain advantages as well as pitfalls that should be acknowledged when considering the best experimental strategy to investigate a particular NCP. In most cases, the combination of several models might be the best option as this can maximize the advantages and reduce the limitations of each individual model. In this regard, it would be beneficial to implement 3-D organoid culture systems (Lancaster and Knoblich, 2014), whereby hESC/hiPSC can be used to more faithfully recapitulate human craniofacial structures *in vitro*.

## Practical Guidelines to Investigate Neurocristopathies Caused by Structural Variants and Involving Long-Range Regulatory Mechanisms

Public repositories offer an increasing amount of functional genomic data obtained from *in vitro*-derived human NCC or early human embryonic tissues with a NC origin, which together represent a highly valuable resource to unravel the etiology of many NCP. Currently, these data sets provide information about gene-expression levels, epigenetic profiles, and enhancer maps in cranial NCC (derived *in vitro*) and craniofacial embryonic tissues (Rada-Iglesias et al., 2012; Prescott et al., 2015; Gerrard et al., 2016; Wilderman et al., 2018; Laugsch et al., 2019). Hence, there is still a clear need for genomic information from more posterior NC types in order to improve our understanding of the full repertoire of human NCP. In principle, gene expression profiles and enhancer maps can be combined with Hi-C data (i.e., TAD maps) in order to identify gene regulatory domains during NC development (as detailed in see section “Pathological Disruption of Regulatory Domains by Structural Variants”). Unfortunately, Hi-C data is still not available for either human NCC or NC-derived embryonic tissues. This will be hopefully solved in the near future because TADs might be more variable among cell types/tissues than previously anticipated (Dixon et al., 2012; McArthur and Capra, 2020). Furthermore, smaller topological domains with important regulatory functions (e.g., sub-TADs) tend to show higher tissue specificity (Berlivet et al., 2013; Kragesteen et al., 2018; Beagan and Phillips-Cremins, 2020). Nevertheless, currently available Hi-C maps (Wang et al., 2018) generated in different human cell types can still be used to infer regulatory domains in the NC. For example, Hi-C maps derived from hESC helped to define the *TFAP2A* regulatory domain in hNCC and to predict the pathomechanism whereby an inversion causes BOFS (Laugsch et al., 2019).

**FIGURE 3:**
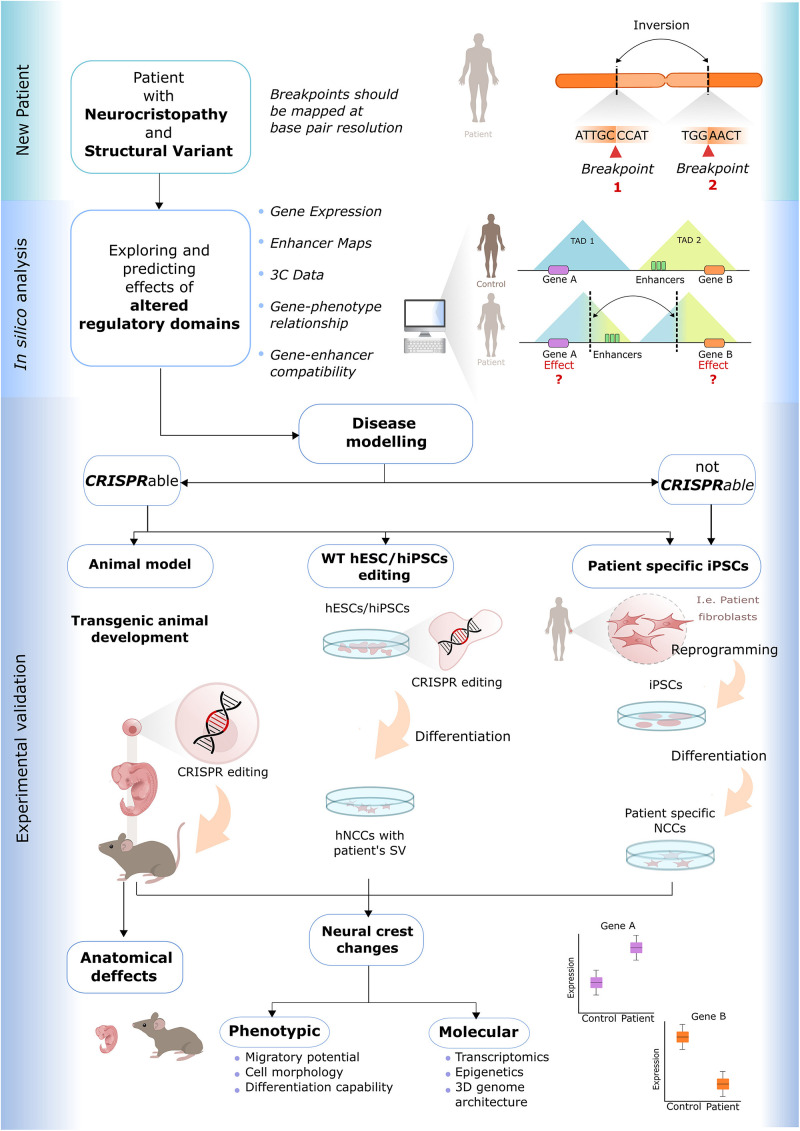
Practical guidelines for the study of human neurocristopathies caused by structural variants. (*New Patient*) These guidelines can be used to study patients with neurocristopathies and structural variants (SV). (*In silico analysis*) The regulatory domains affected by the SV are identified with the use of publicly available genomic data, and the patient etiology can be predicted with the usage of *ad hoc in silico* tools. (*Experimental validation*) Two main experimental validation approaches can be followed. (1) If the SV is amenable to CRISPR-cas engineering (CRISPRable), the disease modeling can be done through (i) genome editing in animal models, if such models display the same gene dosage sensitivity as humans; (ii) genome editing of WT hESC/hiPSC; (iii) derivation of patient-specific hiPSC. (2) If the SV is not amenable to CRISPR-cas engineering (not CRISPRable), the derivation of the patient-specific hiPSCs is the only option. Once the previous disease models are established, a phenotypic and molecular characterization can be performed.

We now provide some practical guidelines that can be used to uncover the pathological mechanisms whereby SVs can cause NCP (Figure 3).

• First, if a patient with a diagnosed NCP and harboring a SV is encountered, it is essential to map the SV breakpoints with base-pair resolution. For this purpose, different methods and tools can be used (Zhao et al., 2013; Ugur Sezerman et al., 2019), such as BreakDancer (Chen et al., 2009), an algorithm for high-resolution mapping of genomic structural variation.
• Then, the functional genomic data (e.g., cell type–specific gene-expression levels, epigenetic profiles, cranial NCCs, and craniofacial embryonic tissue enhancer maps, etc.) described in the previous paragraph can be used to map gene regulatory domains in NC and NC-derived tissues, which, together with *in silico* prediction approaches (as the ones described in see section “Pathological Disruption of Regulatory Domains by Structural Variants”), enable the prediction of the pathological mechanisms causing the NCP. Initially, these approaches can assess if the SV directly disrupt a gene/s and/or if they might involve long-range regulatory mechanisms (e.g., enhancer adoption, enhancer disconnection).
• Next, if any high-confidence pathomechanism is predicted, experimental validations should be carried on. This is especially important in the case of long-range mechanisms due to our still limited capacity to predict the functional relevance of enhancers and to assign them to the correct target genes. Regarding the experimental validation, two main alternatives can be considered:
  (i) SV amenable to engineering with genome editing tools (referred as “CRISPRable” in Figure 3): the patient’s SV can be recapitulated in a model organism, such as mice, to evaluate the molecular and phenotypic consequences during NC development (Lupiáñez et al., 2015; Kragesteen et al., 2018). This requires that the regulatory domain/s potentially disrupted by the SV are evolutionary conserved and that there are no differences in dosage sensitivity for the potentially relevant genes. If these requirements are not fulfilled, the patient’s SV can be introduced into wild-type (WT) hESC/hiPSC that can be then differentiated into NCC and extensively characterized at the molecular (e.g., gene expression, 3-D chromatin structure) and cellular level (e.g., migration, differentiation into NC derivatives). Importantly, both mice and hESC/hiPSC with engineered SV can be compared with isogenic WT controls.
  (ii) SV not (easily) amenable to engineering with genome editing tools (referred to as not CRISPRable in Figure 3): Due to their complexity (multiple breakpoints), very long sizes or type (i.e., translocations), some SV cannot be efficiently engineered using currently available tools. Although technical advances might overcome these limitations in the future (Jiang et al., 2016; Torres-Ruiz et al., 2017), a good alternative to study these SV consists of obtaining patient fibroblasts that can be then reprogrammed into hiPSC (Takahashi et al., 2007). Subsequently, the patient-specific hiPSC can be differentiated into NCC and characterized as described above. This strategy was followed to study the BOFS patient described previously (Laugsch et al., 2019), in which an inversion causes *TFAP2A* haploinsufficiency in NCC by disconnecting one of the *TFAP2A* alleles from its NCC-specific enhancers. One limitation of the use of patient-specific hiPSC is that isogenic WT controls are not readily available. In principle, this could be overcome by repairing the SV, which, unfortunately, might be rather difficult for certain SV, or by using parental controls.

## Conclusion

The study of the NC constitutes an essential step to advance in the comprehension of human development and human congenital disease. Using already available genomic data and various experimental strategies it should be possible to discover new pathological mechanisms causing NCP and involving alterations in long-range gene regulation. The proposed practical guidelines to investigate the pathological consequences of SV can be applied beyond NCP with the ultimate goal of improving the diagnosis, counseling, and even treatment of human congenital disorders.

## Author Contributions

VS-G and AR-I wrote the manuscript. MM-F prepared the figures and corrected the manuscript. All authors contributed to the article and approved the submitted version.

## Conflict of Interest

The authors declare that the research was conducted in the absence of any commercial or financial relationships that could be construed as a potential conflict of interest.

## Footnotes

1 https://www.omim.org/

## Abbreviations

3C: chromosome conformation capture
3-D: three-dimensional
BOFS: branchio-oculo-facial syndrome
CRE: *cis-*regulatory element
hESC: human embryonic stem cell
hIPSC: human induced pluripotent stem cell
NC: neural crest
NCC: neural crest cell
NCP: neurocristopathy
Shh: Sonic hedgehog
SNP: single nucleotide polymorphism
SV: structural variation
TAD: topologically associating domain.

## References

[B1] Akdemir K. C. Le V. T. Chandran S. Li Y. Verhaak R. G. Beroukhim R. (2020). Disruption of chromatin folding domains by somatic genomic rearrangements in human cancer. *Nat. Genet.* 52 294–305. 10.1038/s41588-019-0564-y 32024999 PMC7058537

[B2] Amiel J. Benko S. Gordon C. T. Lyonnet S. (2010). Disruption of long-distance highly conserved noncoding elements in neurocristopathies. *Ann. N. Y. Acad. Sci.* 1214 34–46. 10.1111/j.1749-6632.2010.05878.x 21175683

[B3] Arnold C. D. Zabidi M. A. Pagani M. Rath M. Schernhuber K. Kazmar T. (2017). Genome-wide assessment of sequence-intrinsic enhancer responsiveness at single-base-pair resolution. *Nat. Biotechnol.* 35 136–144. 10.1038/nbt.3739 28024147 PMC5870828

[B4] Bajpai R. Chen D. A. Rada-Iglesias A. Zhang J. Xiong Y. Helms J. (2010). CHD7 cooperates with PBAF to control multipotent neural crest formation. *Nature* 463 958–962. 10.1038/nature08733 20130577 PMC2890258

[B5] Bashkirova E. Lomvardas S. (2019). Olfactory receptor genes make the case for inter-chromosomal interactions. *Curr. Opin. Genet. Dev.* 55 106–113. 10.1016/j.gde.2019.07.004 31491591 PMC6759391

[B6] Beagan J. A. Phillips-Cremins J. E. (2020). On the existence and functionality of topologically associating domains. *Nat. Genet.* 52 8–16. 10.1038/s41588-019-0561-1 31925403 PMC7567612

[B7] Benko S. Fantes J. A. Amiel J. Kleinjan D. J. Thomas S. Ramsay J. (2009). Highly conserved non-coding elements on either side of SOX9 associated with Pierre Robin sequence. *Nat. Genet.* 41 359–364. 10.1038/ng.329 19234473

[B8] Bergeron K.-F. Cardinal T. Touré A. M. Béland M. Raiwet D. L. Silversides D. W. (2015). Male-biased aganglionic megacolon in the tasht mouse line due to perturbation of silencer elements in a large gene desert of chromosome 10. *PLoS Genet.* 11:e1005093. 10.1371/journal.pgen.1005093 25786024 PMC4364714

[B9] Bergeron K. F. Nguyen C. M. A. Cardinal T. Charrier B. Silversides D. W. Pilon N. (2016). Upregulation of the Nr2f1-A830082K12Rik gene pair in murine neural crest cells results in a complex phenotype reminiscent of Waardenburg syndrome type 4. *DMM Dis. Model. Mech.* 9 1283–1293. 10.1242/dmm.026773 27585883 PMC5117235

[B10] Berlivet S. Paquette D. Dumouchel A. Langlais D. Dostie J. Kmita M. (2013). Clustering of tissue-specific sub-TADs accompanies the regulation of hoxa genes in developing limbs. *PLoS Genet.* 9:e1004018. 10.1371/journal.pgen.1004018 24385922 PMC3873244

[B11] Bianco S. Lupiáñez D. G. Chiariello A. M. Annunziatella C. Kraft K. Schöpflin R. (2018). Polymer physics predicts the effects of structural variants on chromatin architecture. *Nat. Genet.* 50 662–667. 10.1038/s41588-018-0098-8 29662163

[B12] Bickmore W. A. (2013). The spatial organization of the human genome. *Annu. Rev. Genomics Hum. Genet.* 14 67–84. 10.1146/annurev-genom-091212-153515 23875797

[B13] Bondurand N. Fouquet V. Baral V. Lecerf L. Loundon N. Goossens M. (2012). Alu-mediated deletion of SOX10 regulatory elements in Waardenburg syndrome type 4. *Eur. J. Hum. Genet.* 20 990–994. 10.1038/ejhg.2012.29 22378281 PMC3421117

[B14] Brewer S. Feng W. Huang J. Sullivan S. Williams T. (2004). Wnt1-Cre-mediated deletion of AP-2α causes multiple neural crest-related defects. *Dev. Biol.* 267 135–152. 10.1016/j.ydbio.2003.10.039 14975722

[B15] Brown R. Feder M. E. (2005). Reverse transcriptional profiling: non-correspondence of transcript level variation and proximal promoter polymorphism. *BMC Genomics* 6:110. 10.1186/1471-2164-6-110 16107220 PMC1192798

[B16] Buecker C. Wysocka J. (2012). Enhancers as information integration hubs in development: lessons from genomics. *Trends Genet.* 28 276–284. 10.1016/j.tig.2012.02.008 22487374 PMC5064438

[B17] Bulger M. Groudine M. (2010). Enhancers: the abundance and function of regulatory sequences beyond promoters. *Dev. Biol.* 339 250–257. 10.1016/j.ydbio.2009.11.035 20025863 PMC3060611

[B18] Bulger M. Groudine M. (2011). Functional and mechanistic diversity of distal transcription enhancers. *Cell* 144 327–339. 10.1016/j.cell.2011.01.024 21295696 PMC3742076

[B19] Chen K. Wallis J. W. McLellan M. D. Larson D. E. Kalicki J. M. Pohl C. S. (2009). BreakDancer: an algorithm for high-resolution mapping of genomic structural variation. *Nat. Methods* 6 677–681. 10.1038/nmeth.1363 19668202 PMC3661775

[B20] Cooper G. M. Coe B. P. Girirajan S. Rosenfeld J. A. Vu T. H. Baker C. (2011). A copy number variation morbidity map of developmental delay. *Nat. Genet.* 43 838–846. 10.1038/ng.909 21841781 PMC3171215

[B21] Cordero D. R. Brugmann S. Chu Y. Bajpai R. Jame M. Helms J. A. (2011). Cranial neural crest cells on the move: their roles in craniofacial development. *Am. J. Med. Genet. Part A* 155 270–279. 10.1002/ajmg.a.33702 21271641 PMC3039913

[B22] Dixon J. R. Selvaraj S. Yue F. Kim A. Li Y. Shen Y. (2012). Topological domains in mammalian genomes identified by analysis of chromatin interactions. *Nature* 485 376–380. 10.1038/nature11082 22495300 PMC3356448

[B23] Doni Jayavelu N. Jajodia A. Mishra A. Hawkins R. D. (2020). Candidate silencer elements for the human and mouse genomes. *Nat. Commun.* 11 1–15. 10.1038/s41467-020-14853-5 32103011 PMC7044160

[B24] Elgar G. Vavouri T. (2008). Tuning in to the signals: noncoding sequence conservation in vertebrate genomes. *Trends Genet.* 24 344–352. 10.1016/j.tig.2008.04.005 18514361

[B25] Emison E. S. McCallion A. S. Kashuk C. S. Bush R. T. Grice E. Lin S. (2005). A common sex-dependent mutation in a RET enhancer underlies Hirschsprung disease risk. *Nature* 434 857–863. 10.1038/nature03467 15829955

[B26] Fattahi F. Steinbeck J. A. Kriks S. Tchieu J. Zimmer B. Kishinevsky S. (2016). Deriving human ENS lineages for cell therapy and drug discovery in Hirschsprung disease. *Nature* 531 105–109. 10.1038/nature16951 26863197 PMC4846424

[B27] Frith T. J. Granata I. Wind M. Stout E. Thompson O. Neumann K. (2018). Human axial progenitors generate trunk neural crest cells in vitro. *eLife* 7:e35786. 10.7554/eLife.35786 30095409 PMC6101942

[B28] Galupa R. Pierre Nora E. Worsley-Hunt R. Ohler U. Giorgetti L. Heard Correspondence E. (2020). A conserved noncoding locus regulates random monoallelic xist expression across a topological boundary. *Mol. Cell* 77 1–16. 10.1016/j.molcel.2019.10.030 31759823 PMC6964159

[B29] Ganel L. Abel H. J. Hall I. M. (2017). SVScore: an impact prediction tool for structural variation. *Bioinformatics* 33 1083–1085. 10.1093/bioinformatics/btw789 28031184 PMC5408916

[B30] Gaszner M. Felsenfeld G. (2006). Insulators: exploiting transcriptional and epigenetic mechanisms. *Nat. Rev. Genet.* 7 703–713. 10.1038/nrg1925 16909129

[B31] Gerrard D. T. Berry A. A. Jennings R. E. Piper Hanley K. Bobola N. Hanley N. A. (2016). An integrative transcriptomic atlas of organogenesis in human embryos. *eLife* 5:e15657. 10.7554/eLife.15657 27557446 PMC4996651

[B32] Ghavi-Helm Y. (2019). Functional consequences of chromosomal rearrangements on gene expression: not so deleterious after all? *J. Mol. Biol.* 432 665–675. 10.1016/j.jmb.2019.09.010 31626801

[B33] Ghavi-Helm Y. Jankowski A. Meiers S. Viales R. R. Korbel J. O. Furlong E. E. M. (2019). Highly rearranged chromosomes reveal uncoupling between genome topology and gene expression. *Nat. Genet.* 51 1272–1282. 10.1038/s41588-019-0462-3 31308546 PMC7116017

[B34] Green S. A. Simoes-Costa M. Bronner M. E. (2015). Evolution of vertebrates as viewed from the crest. *Nature* 520 474–482. 10.1038/nature14436 25903629 PMC5100666

[B35] Haberle V. Arnold C. D. Pagani M. Rath M. Schernhuber K. Stark A. (2019). Transcriptional cofactors display specificity for distinct types of core promoters. *Nature* 570 122–126. 10.1038/s41586-019-1210-7 31092928 PMC7613045

[B36] Hackland J. O. S. Frith T. J. R. Thompson O. Marin Navarro A. Garcia-Castro M. I. Unger C. (2017). Top-down inhibition of BMP signaling enables robust induction of hPSCs into neural crest in fully defined, xeno-free conditions. *Stem Cell Rep.* 9 1043–1052. 10.1016/j.stemcr.2017.08.008 28919261 PMC5639211

[B37] Heintzman N. D. Hon G. C. Hawkins R. D. Kheradpour P. Stark A. Harp L. F. (2009). Histone modifications at human enhancers reflect global cell-type-specific gene expression. *Nature* 459 108–112. 10.1038/nature07829 19295514 PMC2910248

[B38] Hertzberg J. Mundlos S. Vingron M. Gallone G. (2020). TADA – a machine learning tool for functional annotation based prioritisation of putative pathogenic CNVs. *bioRxiv* [Preprint]. 10.1101/2020.06.30.180711 PMC8886976 35232478

[B39] Ho S. S. Urban A. E. Mills R. E. (2020). Structural variation in the sequencing era. *Nat. Rev. Genet.* 21 171–189. 10.1038/s41576-019-0180-9 31729472 PMC7402362

[B40] Huang M. Miller M. L. McHenry L. K. Zheng T. Zhen Q. Ilkhanizadeh S. (2016). Generating trunk neural crest from human pluripotent stem cells. *Sci. Rep.* 6:19727. 10.1038/srep19727 26812940 PMC4728437

[B41] Ibn-Salem J. Köhler S. Love M. I. Chung H.-R. Huang N. Hurles M. E. (2014). Deletions of chromosomal regulatory boundaries are associated with congenital disease. *Genome Biol.* 15:423. 10.1186/s13059-014-0423-1 25315429 PMC4180961

[B42] Jiang J. Zhang L. Zhou X. Chen X. Huang G. Li F. (2016). Induction of site-specific chromosomal translocations in embryonic stem cells by CRISPR/Cas9. *Sci. Rep.* 6:21918. 10.1038/srep21918 26898344 PMC4761995

[B43] Kleinjan D. A. van Heyningen V. (2005). Long-range control of gene expression: emerging mechanisms and disruption in disease. *Am. J. Hum. Genet.* 76 8–32. 10.1086/426833 15549674 PMC1196435

[B44] Kraft K. Magg A. Heinrich V. Riemenschneider C. Schöpflin R. Markowski J. (2019). Serial genomic inversions induce tissue-specific architectural stripes, gene misexpression and congenital malformations. *Nat. Cell Biol.* 21 305–310. 10.1038/s41556-019-0273-x 30742094

[B45] Kragesteen B. K. Spielmann M. Paliou C. Heinrich V. Schöpflin R. Esposito A. (2018). Dynamic 3D chromatin architecture contributes to enhancer specificity and limb morphogenesis. *Nat. Genet.* 50 1463–1473. 10.1038/s41588-018-0221-x 30262816 PMC10154999

[B46] Krijger P. H. L. De Laat W. (2016). Regulation of disease-associated gene expression in the 3D genome. *Nat. Rev. Mol. Cell Biol.* 17 771–782. 10.1038/nrm.2016.138 27826147

[B47] Lam M. T. Y. Li W. Rosenfeld M. G. Glass C. K. (2014). Enhancer RNAs and regulated transcriptional programs. *Trends Biochem. Sci.* 39 170–182. 10.1016/j.tibs.2014.02.007 24674738 PMC4266492

[B48] Lancaster M. A. Knoblich J. A. (2014). Organogenesis in a dish: modeling development and disease using organoid technologies. *Science* 345:1247125. 10.1126/science.1247125 25035496

[B49] Laugsch M. Bartusel M. Rehimi R. Alirzayeva H. Karaolidou A. Crispatzu G. (2019). Modeling the pathological long-range regulatory effects of human structural variation with patient-specific hiPSCs. *Cell Stem Cell* 24 736.e12–752.e12. 10.1016/j.stem.2019.03.004 30982769

[B50] Leblanc S. K. Yu S. Barnett C. P. (2013). 6p.24 microdeletion involving TFAP2A without classic features of branchio-oculo-facial syndrome. *Am. J. Med. Genet. Part A* 161 901–904. 10.1002/ajmg.a.35804 23495225

[B51] Lecerf L. Kavo A. Ruiz-Ferrer M. Baral V. Watanabe Y. Chaoui A. (2014). An impairment of long distance SOX10 regulatory elements underlies isolated hirschsprung disease. *Hum. Mutat.* 35 303–307. 10.1002/humu.22499 24357527

[B52] Lettice L. A. (2003). A long-range Shh enhancer regulates expression in the developing limb and fin and is associated with preaxial polydactyly. *Hum. Mol. Genet.* 12 1725–1735. 10.1093/hmg/ddg180 12837695

[B53] Lettice L. A. Daniels S. Sweeney E. Venkataraman S. Devenney P. S. Gautier P. (2011). Enhancer-adoption as a mechanism of human developmental disease. *Hum. Mutat.* 32 1492–1499. 10.1002/humu.21615 21948517

[B54] Li H. Sheridan R. Williams T. (2013). Analysis of TFAP2A mutations in branchio-oculo-facial syndrome indicates functional complexity within the AP-2α DNA-binding domain. *Hum. Mol. Genet.* 22 3195–3206. 10.1093/hmg/ddt173 23578821 PMC3723307

[B55] Lieberman-Aiden E. van Berkum N. L. Williams L. Imakaev M. Ragoczy T. Telling A. (2009). Comprehensive mapping of long-range interactions reveals folding principles of the human genome. *Science* 326 289–293. 10.1126/science.1181369 19815776 PMC2858594

[B56] Lupiáñez D. G. Kraft K. Heinrich V. Krawitz P. Brancati F. Klopocki E. (2015). Disruptions of topological chromatin domains cause pathogenic rewiring of gene-enhancer interactions. *Cell* 161 1012–1025. 10.1016/j.cell.2015.04.004 25959774 PMC4791538

[B57] Lupiáñez D. G. Spielmann M. Mundlos S. (2016). Breaking TADs: how alterations of chromatin domains result in disease. *Trends Genet.* 32 225–237. 10.1016/j.tig.2016.01.003 26862051

[B58] Maurano M. T. Humbert R. Rynes E. Thurman R. E. Haugen E. Wang H. (2012). Systematic localization of common disease-associated variation in regulatory DNA. *Science* 337 1190–1195. 10.1126/science.1222794 22955828 PMC3771521

[B59] McArthur E. Capra J. A. (2020). Topologically associating domain (TAD) boundaries stable across diverse cell types are evolutionarily constrained and enriched for heritability. *bioRxiv* 2020.01.10.901967 10.1101/2020.01.10.901967 PMC7895846 33545030

[B60] McLaren W. Gil L. Hunt S. E. Riat H. S. Ritchie G. R. S. Thormann A. (2016). The ensembl variant effect predictor. *Genome Biol.* 17:122. 10.1186/s13059-016-0974-4 27268795 PMC4893825

[B61] Menendez L. Kulik M. J. Page A. T. Park S. S. Lauderdale J. D. Cunningham M. L. (2013). Directed differentiation of human pluripotent cells to neural crest stem cells. *Nat. Protoc.* 8 203–212. 10.1038/nprot.2012.156 23288320

[B62] Mestas J. Hughes C. C. W. (2004). Of mice and not men: differences between mouse and human immunology. *J. Immunol.* 172 2731–2738. 10.4049/jimmunol.172.5.2731 14978070

[B63] Mica Y. Lee G. Chambers S. M. Tomishima M. J. Studer L. (2013). Modeling neural crest induction, melanocyte specification, and disease-related pigmentation defects in hESCs and patient-specific iPSCs. *Cell Rep.* 3 1140–1152. 10.1016/j.celrep.2013.03.025 23583175 PMC3681528

[B64] Middelkamp S. Vlaar J. M. Giltay J. Korzelius J. Besselink N. Boymans S. (2019). Prioritization of genes driving congenital phenotypes of patients with de novo genomic structural variants. *Genome Med.* 11:79. 10.1186/s13073-019-0692-0 31801603 PMC6894143

[B65] Milunsky J. M. Maher T. A. Zhao G. Roberts A. E. Stalker H. J. Zori R. T. (2008). TFAP2A mutations result in branchio-oculo-facial syndrome. *Am. J. Hum. Genet.* 82 1171–1177. 10.1016/j.ajhg.2008.03.005 18423521 PMC2427243

[B66] Milunsky J. M. Maher T. M. Zhao G. Wang Z. Mulliken J. B. Chitayat D. (2011). Genotype-phenotype analysis of the branchio-oculo-facial syndrome. *Am. J. Med. Genet. Part A* 155 22–32. 10.1002/ajmg.a.33783 21204207

[B67] Monahan K. Horta A. Lomvardas S. (2019). LHX2- and LDB1-mediated trans interactions regulate olfactory receptor choice. *Nature* 565 448–453. 10.1038/s41586-018-0845-0 30626972 PMC6436840

[B68] Müller H. P. Schaffner W. (1990). Transcriptional enhancers can act in trans. *Trends Genet.* 6 300–304. 10.1016/0168-9525(90)90236-Y 2238088

[B69] Mumbach M. R. Rubin A. J. Flynn R. A. Dai C. Khavari P. A. Greenleaf W. J. (2016). HiChIP: efficient and sensitive analysis of protein-directed genome architecture. *Nat. Methods* 13 919–922. 10.1038/nmeth.3999 27643841 PMC5501173

[B70] Ngan C. Y. Wong C. H. Tjong H. Wang W. Goldfeder R. L. Choi C. (2020). Chromatin interaction analyses elucidate the roles of PRC2-bound silencers in mouse development. *Nat. Genet.* 52 264–272. 10.1038/s41588-020-0581-x 32094912 PMC7869692

[B71] Nora E. P. Dekker J. Heard E. (2013). Segmental folding of chromosomes: a basis for structural and regulatory chromosomal neighborhoods? *BioEssays* 35 818–828. 10.1002/bies.201300040 23832846 PMC3874840

[B72] Nora E. P. Goloborodko A. Valton A. L. Gibcus J. H. Uebersohn A. Abdennur N. (2017). Targeted degradation of CTCF decouples local insulation of chromosome domains from genomic compartmentalization. *Cell* 169 930.e4–944.e4. 10.1016/j.cell.2017.05.004 28525758 PMC5538188

[B73] Ong C.-T. Corces V. G. (2011). Enhancer function: new insights into the regulation of tissue-specific gene expression. *Nat. Rev. Genet.* 12 283–293. 10.1038/nrg2957 21358745 PMC3175006

[B74] Osterwalder M. Barozzi I. Tissiéres V. Fukuda-Yuzawa Y. Mannion B. J. Afzal S. Y. (2018). Enhancer redundancy provides phenotypic robustness in mammalian development. *Nature* 554 239–243. 10.1038/nature25461 29420474 PMC5808607

[B75] Palstra R.-J. Tolhuis B. Splinter E. Nijmeijer R. Grosveld F. de Laat W. (2003). The β-globin nuclear compartment in development and erythroid differentiation. *Nat. Genet.* 35 190–194. 10.1038/ng1244 14517543

[B76] Pang B. Snyder M. P. (2020). Systematic identification of silencers in human cells. *Nat. Genet.* 52 254–263. 10.1038/s41588-020-0578-5 32094911 PMC7148122

[B77] Prescott S. L. Srinivasan R. Marchetto M. C. Grishina I. Narvaiza I. Selleri L. (2015). Enhancer Divergence and cis-regulatory evolution in the human and chimp neural crest. *Cell* 163 68–83. 10.1016/J.CELL.2015.08.036 26365491 PMC4848043

[B78] ENCODE Project Consortium (2012). An integrated encyclopedia of DNA elements in the human genome. *Nature* 489 57–74. 10.1038/nature11247 22955616 PMC3439153

[B79] Rada-Iglesias A. Bajpai R. Prescott S. Brugmann S. A. Swigut T. Wysocka J. (2012). Epigenomic annotation of enhancers predicts transcriptional regulators of human neural crest. *Cell Stem Cell* 11, 633–648. 10.1016/j.stem.2012.07.006 22981823 PMC3751405

[B80] Rada-Iglesias A. Bajpai R. Swigut T. Brugmann S. A. Flynn R. A. Wysocka J. (2011). A unique chromatin signature uncovers early developmental enhancers in humans. *Nature* 470 279–283. 10.1038/nature09692 21160473 PMC4445674

[B81] Rada-Iglesias A. Wysocka J. (2011). Epigenomics of human embryonic stem cells and induced pluripotent stem cells: insights into pluripotency and implications for disease. *Genome Med.* 3:36. 10.1186/gm252 21658297 PMC3218810

[B82] Rahimov F. Jugessur A. Murray J. C. (2012). Genetics of nonsyndromic orofacial clefts. *Cleft Palate-Craniof. J.* 49 73–91. 10.1597/10-178 21545302 PMC3437188

[B83] Rao S. S. P. Huang S. C. Glenn St Hilaire B. Engreitz J. M. Perez E. M. (2017). Cohesin loss eliminates all loop domains. *Cell* 171 305.e24–320.e24. 10.1016/j.cell.2017.09.026 28985562 PMC5846482

[B84] Rao S. S. P. Huntley M. H. Durand N. C. Stamenova E. K. Bochkov I. D. Robinson J. T. (2014). A 3D map of the human genome at kilobase resolution reveals principles of chromatin looping. *Cell* 159 1665–1680. 10.1016/j.cell.2014.11.021 25497547 PMC5635824

[B85] Redin C. Brand H. Collins R. L. Kammin T. Mitchell E. Hodge J. C. (2017). The genomic landscape of balanced cytogenetic abnormalities associated with human congenital anomalies. *Nat. Genet.* 49 36–45. 10.1038/ng.3720 27841880 PMC5307971

[B86] Sagai T. Hosoya M. Mizushina Y. Tamura M. Shiroishi T. (2005). Elimination of a long-range cis-regulatory module causes complete loss of limb-specific Shh expression and truncation of the mouse limb. *Development* 132 797–803. 10.1242/dev.01613 15677727

[B87] Sanyal A. Lajoie B. R. Jain G. Dekker J. (2012). The long-range interaction landscape of gene promoters. *Nature* 489 109–113. 10.1038/nature11279 22955621 PMC3555147

[B88] Sauka-Spengler T. Bronner-Fraser M. (2008). A gene regulatory network orchestrates neural crest formation. *Nat. Rev. Mol. Cell Biol.* 9 557–568. 10.1038/nrm2428 18523435

[B89] Savarese F. Grosschedl R. (2006). Blurring cis and trans in gene regulation. *Cell* 126 248–250. 10.1016/j.cell.2006.07.008 16873057

[B90] Schorle H. Meier P. Buchert M. Jaenisch R. Mitchell P. J. (1996). Transcription factor AP-2 essential for cranial closure and craniofacial development. *Nature* 381 235–238. 10.1038/381235a0 8622765

[B91] Sebat J. Lakshmi B. Malhotra D. Troge J. Lese-Martin C. Walsh T. (2007). Strong association of de novo copy number mutations with autism. *Science* 316 445–449. 10.1126/science.1138659 17363630 PMC2993504

[B92] Simões-Costa M. Bronner M. E. (2013). Insights into neural crest development and evolution from genomic analysis. *Genome Res.* 23 1069–1080. 10.1101/gr.157586.113 23817048 PMC3698500

[B93] Soemedi R. Wilson I. J. Bentham J. Darlay R. Töpf A. Zelenika D. (2012). Contribution of global rare copy-number variants to the risk of sporadic congenital heart disease. *Am. J. Hum. Genet.* 91 489–501. 10.1016/j.ajhg.2012.08.003 22939634 PMC3511986

[B94] Spielmann M. Lupiáñez D. G. Mundlos S. (2018). Structural variation in the 3D genome. *Nat. Rev. Genet.* 19 453–467. 10.1038/s41576-018-0007-0 29692413

[B95] Sudmant P. H. Rausch T. Gardner E. J. Handsaker R. E. Abyzov A. Huddleston J. (2015). An integrated map of structural variation in 2,504 human genomes. *Nature* 526 75–81. 10.1038/nature15394 26432246 PMC4617611

[B96] Symmons O. Pan L. Remeseiro S. Aktas T. Klein F. Huber W. (2016). The Shh topological domain facilitates the action of remote enhancers by reducing the effects of genomic distances. *Dev. Cell* 39 529–543. 10.1016/j.devcel.2016.10.015 27867070 PMC5142843

[B97] Takahashi K. Tanabe K. Ohnuki M. Narita M. Ichisaka T. Tomoda K. (2007). Induction of pluripotent stem cells from adult human fibroblasts by defined factors. *Cell* 131 861–872. 10.1016/j.cell.2007.11.019 18035408

[B98] Tchieu J. Zimmer B. Fattahi F. Amin S. Zeltner N. Chen S. (2017). A modular platform for differentiation of human PSCs into all major ectodermal lineages. *Cell Stem Cell* 21 399.e–410.e. 10.1016/j.stem.2017.08.015 28886367 PMC5737635

[B99] Torres-Ruiz R. Martinez-Lage M. Martin M. C. Garcia A. Bueno C. Castaño J. (2017). Efficient Recreation of t(11;22) EWSR1-FLI1+ in human stem cells using CRISPR/Cas9. *Stem Cell Rep.* 8 1408–1420. 10.1016/j.stemcr.2017.04.014 28494941 PMC5425785

[B100] Trainor P. A. (2010). Craniofacial birth defects: the role of neural crest cells in the etiology and pathogenesis of Treacher Collins syndrome and the potential for prevention. *Am. J. Med. Genet. Part A* 152A 2984–2994. 10.1002/ajmg.a.33454 20734335 PMC3686507

[B101] Ugur Sezerman O. Ulgen E. Seymen N. Melis Durasi I. (2019). “Bioinformatics workflows for genomic variant discovery, interpretation and prioritization,” in *Bioinformatics Tools for Detection and Clinical Interpretation of Genomic Variations*, eds Samadikuchaksaraei A. Seifi M. (London: IntechOpen).

[B102] van de Werken H. J. G. de Vree P. J. P. Splinter E. Holwerda S. J. B. Klous P. de Wit E. (2012). 4C technology: protocols and data analysis. *Methods Enzymol.* 513 89–112. 10.1016/B978-0-12-391938-0.00004-5 22929766

[B103] Vega-Lopez G. A. Cerrizuela S. Tribulo C. Aybar M. J. (2018). Neurocristopathies: new insights 150 years after the neural crest discovery. *Dev. Biol.* 444 S110–S143. 10.1016/j.ydbio.2018.05.013 29802835

[B104] Walsh T. McClellan J. M. McCarthy S. E. Addington A. M. Pierce S. B. Cooper G. M. (2008). Rare structural variants disrupt multiple genes in neurodevelopmental pathways in schizophrenia. *Science* 320 539–543. 10.1126/science.1155174 18369103

[B105] Wang Y. Song F. Zhang B. Zhang L. Xu J. Kuang D. (2018). The 3D genome browser: a web-based browser for visualizing 3D genome organization and long-range chromatin interactions. *Genome Biol.* 19:151. 10.1186/s13059-018-1519-9 30286773 PMC6172833

[B106] Weischenfeldt J. Dubash T. Drainas A. P. Mardin B. R. Chen Y. Stütz A. M. (2017). Pan-cancer analysis of somatic copy-number alterations implicates IRS4 and IGF2 in enhancer hijacking. *Nat. Genet.* 49 65–74. 10.1038/ng.3722 27869826 PMC5791882

[B107] Wilderman A. VanOudenhove J. Kron J. Noonan J. P. Cotney J. (2018). High-resolution epigenomic atlas of human embryonic craniofacial development. *Cell Rep.* 23 1581–1597. 10.1016/j.celrep.2018.03.129 29719267 PMC5965702

[B108] Wittkopp P. J. Kalay G. (2012). Cis-regulatory elements: molecular mechanisms and evolutionary processes underlying divergence. *Nat. Rev. Genet.* 13 59–69. 10.1038/nrg3095 22143240

[B109] Wray G. A. (2007). The evolutionary significance of cis-regulatory mutations. *Nat. Rev. Genet.* 8 206–216. 10.1038/nrg2063 17304246

[B110] Xu B. Roos J. L. Levy S. van Rensburg E. J. Gogos J. A. Karayiorgou M. (2008). Strong association of de novo copy number mutations with sporadic schizophrenia. *Nat. Genet.* 40 880–885. 10.1038/ng.162 18511947

[B111] Yang L. Luquette L. J. Gehlenborg N. Xi R. Haseley P. S. Hsieh C.-H. (2013). Diverse Mechanisms of somatic structural variations in human cancer genomes. *Cell* 153 919–929. 10.1016/j.cell.2013.04.010 23663786 PMC3704973

[B112] Yauy K. Gatinois V. Guignard T. Sati S. Puechberty J. Gaillard J. B. (2018). Looking for broken TAD boundaries and changes on DNA interactions: clinical guide to 3D chromatin change analysis in complex chromosomal rearrangements and chromothripsis. *Methods Mol.* 1769 353–361. 10.1007/978-1-4939-7780-2_22 29564835

[B113] Zepeda-Mendoza C. J. Ibn-Salem J. Kammin T. Harris D. J. Rita D. Gripp K. W. (2017). Computational prediction of position effects of apparently balanced human chromosomal rearrangements. *Am. J. Hum. Genet.* 101 206–217. 10.1016/j.ajhg.2017.06.011 28735859 PMC5544382

[B114] Zhang J. Hagopian-Donaldson S. Serbedzija G. Elsemore J. Plehn-Dujowich D. McMahon A. P. (1996). Neural tube, skeletal and body wall defects in mice lacking transcription factor AP-2. *Nature* 381 238–241. 10.1038/381238a0 8622766

[B115] Zhao M. Wang Q. Wang Q. Jia P. Zhao Z. (2013). Computational tools for copy number variation (CNV) detection using next-generation sequencing data: features and perspectives. *BMC Bioinform.* 14:S1. 10.1186/1471-2105-14-S11-S1 24564169 PMC3846878

